# Transformation from Quantum to Classical Mode: the Size Effect of Plasmon in 2D Atomic Cluster System

**DOI:** 10.1038/s41598-019-43249-9

**Published:** 2019-04-29

**Authors:** Reng-lai Wu, Jun Quan, Chunhua Tian, Mengtao Sun

**Affiliations:** 1 School of Physical Science and Technology, Lingnan Normal University, Zhanjiang, 524048 P.R. China; 2 School of Mathematics and Physics, Beijing Key Laboratory for Magneto-Photoelectrical Composite and Interface Science, University of Science and Technology Beijing, Beijing, 100083 P.R. China

**Keywords:** Nanophotonics and plasmonics, Nanophotonics and plasmonics, Nanophotonics and plasmonics

## Abstract

On the basis of tight-binding approximation, the energy absorption of 2D atomic clusters is calculated by the linear response theory. Through the energy-absorption peaks in the presence of different external potentials, various types of plasmon modes are specified in clusters with dozens to hundreds atoms, such as transverse dipole plasmon, longitudinal dipole plasmon, transverse quadrupole plasmon, and longitudinal quadrupole plasmon. Moreover, the transformation of plasmon from quantum to classical mode is observed in clusters with different shape and different electron density. The particular transformation process demonstrate that: there are only a few modes of plasmon in clusters with few-atoms; as the number of atoms in cluster is increased, the number of plasmon modes increases, the gaps between plasmon frequencies become smaller, the quantum modes of plasmon gradually evolve into continuous modes, and the dispersion of quantum-mode plasmon gradually transforms into the one of classical 2D plasmon. Such process reveals the size effect of plasmon in 2D clusters, which can be explained by the fact that the energy levels near the Fermi energy are denser and more compact in larger-size clusters.

## Introduction

With the development of nanotechnology and the miniaturization of optoelectronic devices, it has becomes increasingly necessary to explore and better understand the excitation properties of plasmon in nanoclusters. For example, the plasmon biosensor is applied to detect cell pathological changes according to the sensitive interactions between light and nano-scale biological molecules^1^. On the other hand, the investigation of the plasmon at the nanoscale is motivated by its applications in nanotechnology and biotherapy areas^2–8^. Among the researches on plasmon in nano-structures, classical theory begins to fail in micro system with size less than 10 nanometers, while the quantum effect of plasmon comes to play an important role. which may lead to some distinctive and unusual properties, such as the change of plasmon’s frequency and lifetime^9–12^, the blueshifts and redshifts of plasmons’ resonances with the increase of nano-system size^13–17^, and different plasmon modes under different electric potentials^18–21^. Much research work has been done to understand the quantum effects of plasmon. Scholl and co-workers used an analytical quantum mechanical model to describe the shift of plasmon frequency^10^. Nordlander’s team proposed a quantum dynamic scheme to research the influence of quantum effect on the plasma resonance in two separated nanodimers, and found that, as the distance between the two nanodimers is less than 1 nanometer, the enhancement of electromagnetic field would be much smaller than the result predicted by the classical theory^11^. Applying the time-dependent density functional theory and local density approximation, Gao’s team found the transverse and longitudinal plasmon in linear metallic atomic chain, which excited by the electric field along transverse and longitudinal direction, respectively^12–14^. Basing on the linear-response theory and the eigen-equation method, Bertsch, Yu, Xue, *et al*. found lots of plasmon modes in various atomic clusters^18–20^, interestingly, the earliar experiment carried out by Schlipper had already reported the multiple collective plasmon resonance in the sodium cluster^21^. These studies provide meaningful results for the quantum effect of plasmon, and a lot of them have demonstrated the presence of discrete quantum modes of plasmon in nano-cluster system^12–21^. However, the dispersion of quantum-mode plasmon in nano-cluster system is still indistinct, and the transformation process of the plasmon from quantum modes to the classical mode is still unknown.

In this paper, we apply the linear response theory to calculate the energy absorption of plasmon in two dimensional (2D) atomic cluster system. The results reveal that quantum modes of plasmon exist in 2D atomic cluster system. However, the size effect of plasmon arises with changing cluster dimensions and electron density, resulting in a transformation of the quantum-mode plasmon into the classical one as the cluster dimensions grow.

## Model and Theory

**Figure 1:**
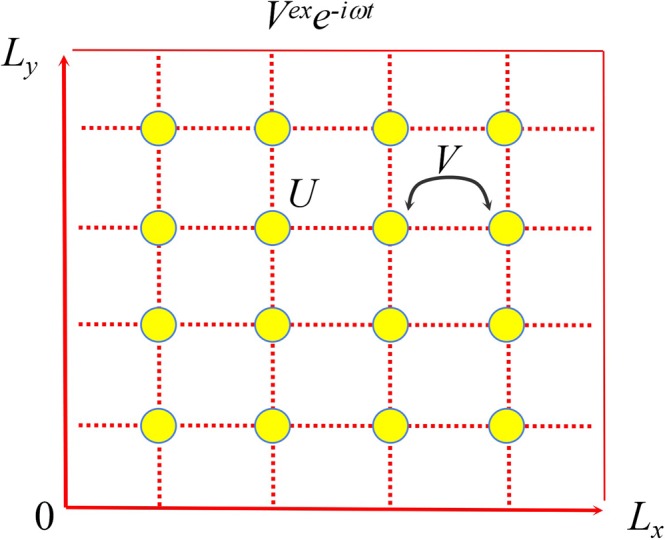
The model of 2D atomic cluster with size *N*_*x*_ × *N*_*y*_ = 4 × 4.

The model of 2D atomic cluster under study is presented in Fig. 1, where the transverse length *L*_*x*_ = (*N*_*x*_ + 1)*a*, and the longitudinal length *L*_*y*_ = (*N*_*y*_ + 1)*a*. Here, *N*_*x*_ and *N*_*y*_, respectively, are the atom numbers in the transverse and longitudinal directions, *a* is the distance between the nearest two atoms. *V*^*ex*^(***l****, ω*)*e*^−*iωt*^ is the time-dependent external electric potential, where, *V*^*ex*^(***l****, ω*) is the space distribution of the external potential, ***l*** = *l*_*x*_***e***_***x***_ + *l*_*y*_***e***_***y***_ is the lattice coordinate, *l*_*x*_ and *l*_*y*_ respectively are components of ***l*** in *X* and *Y* directions, ***e***_***x***_ and ***e***_*y*_ respectively are unit vectors in *X* and *Y* directions, and *ω* the frequency of the external potential. *U* is the on-site Coulomb interaction, and *V* is the nearest-neighbor Coulomb interactions^22,23^. In this model, electrons are tightly bound to the lattice points, and can only hop into neighboring lattice points, so the model is suitable for crystals whose lattice constant is much larger than the atom radius.

In the wannier representation, the plasmon excited by *V*^*ex*^(***l****, ω*)*e*^−*iωt*^ can be found by the peaks of energy absorption function:

1 $$L(\omega )=\frac{1}{2}\omega \,{\rm{Im}}\{\sum _{{\bf{l}}}[{V}^{ex}(l,\omega )]\,Q(l,\omega )\}$$

where *Q*(***l***, *ω*) is the induced charge. Based on the linear response theory and tight- binding approximation, *Q*(***l***, *ω*) can be determined by^17,19^

2 $$\sum _{{\boldsymbol{l}}^{\prime} }[{\delta }_{ll^{\prime} }-\sum _{{{\boldsymbol{l}}}^{^{\prime\prime} }}{\rm{\Pi }}({\boldsymbol{l}},{{\boldsymbol{l}}}^{^{\prime\prime} },\omega ){v}_{{\boldsymbol{l}}^{\prime\prime} {\boldsymbol{l}}^{\prime} }]Q({\boldsymbol{l}}^{\prime} ,\omega )={e}^{2}\sum _{{\boldsymbol{l}}^{\prime} }{\rm{\Pi }}({\bf{l}},{\boldsymbol{l}}^{\prime} ,\omega ){V}^{ex}({\boldsymbol{l}}^{\prime} ,\omega )$$

Here, *v*_***l***″***l***′_ is given by

3 $${v}_{{{\boldsymbol{l}}}^{^{\prime\prime} }{\boldsymbol{l}}^{\prime} }=\{\begin{array}{cc}\frac{U}{2}, & {\boldsymbol{l}}^{\prime} ={{\boldsymbol{l}}}^{^{\prime\prime} }\\ V, & |{\boldsymbol{l}}^{\prime} -{{\boldsymbol{l}}}^{^{\prime\prime} }|=a\\ 0, & {\rm{el}}\,{\rm{se}}\end{array}$$

and Π(***l***, ***l***′, *ω*) is the Linhard function:

4 $${\rm{\Pi }}({\boldsymbol{l}},{\boldsymbol{l}}^{\prime} ,\omega )=2\sum _{mn}\frac{f({E}_{m})-f({E}_{n})}{{E}_{m}-{E}_{n}-\omega -i\eta }{\psi }_{m}^{\ast }({\boldsymbol{l}}){\psi }_{n}({\boldsymbol{l}}){\psi }_{n}^{\ast }({\boldsymbol{l}}^{\prime} ){\psi }_{m}({\boldsymbol{l}}^{\prime} )$$

where, *f*(*E*_*m*_) is the Fermi function, *η* is the scattering rate, and ${\psi }_{n}({\bf{l}})$ is the eigenvector corresponding to eigenvalue *E*_*n*_, which can be written as

5 $${\psi }_{n}({\boldsymbol{l}})=\sqrt{\frac{4}{{L}_{x}{L}_{y}}}\,\sin (\frac{{n}_{x}\pi }{{L}_{x}}{l}_{x})(\frac{{n}_{y}\pi }{{L}_{y}}{l}_{y})$$

6 $${E}_{n}=-2\gamma \,\cos (\frac{{n}_{x}\pi }{{L}_{x}})\,-\,2\gamma \,\cos (\frac{{n}_{y}\pi }{{L}_{y}})$$

where, *n*_*x*_ = 1, 2, 3, …. *N*_*x*_, *n*_*y*_ = 1, 2, 3, …. *N*_*y*_. *γ* is the nearest-neighbor transfer energy.

## Results and Discussions

In all the calculations, we set the parameters that *U* = 3.0 *e*V, *V* = 1.0 *e*V, $\eta =0.005$ *e*V. The unit of frequency is $\gamma /\hslash$, with $\hslash$ the Planck’s constant, and that of the space coordinate is the distance *a*, with *a* = 0.4 nm. The size of the system is expressed by *N*_*x*_ × *N*_*y*_, the face electron density *n*_*e*_ = *N*_*e*_*/*(*N*_*x*_ *N*_*y*_*a*^2^), where *N*_*e*_ is the electron number. *V*_*L*_^*ex*^ is the potential of longitudinal electric field which is applied in the *y* direction; *V*_*T*_^*ex*^ is the potential of transverse electric field which is applied in the *x* direction. Arbitrary units are adopted for the values of the energy absorption and the charge.

**Figure 2:**
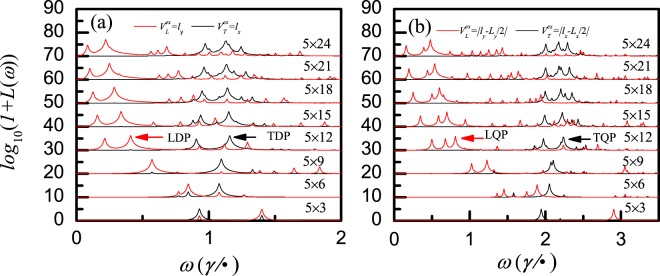
The evolution of energy absorption with the longitudinal size *N*_*y*_ for a fixed transverse size *N*_*x*_ = 5, whereas *N*_*y*_ = 3, 6, 9, 12, 15, 18, 21, 24 from bottom to top. (**a**) The applied external potentials are *V*_*T*_^*ex*^ = *l*_*x*_ and *V*_*L*_^*ex*^ = *l*_*y*_; (**b**) The applied external potentials are *V*_*T*_^*ex*^ = |*l*_*x*_−*L*_*x*_/2| and *V*_*L*_^*ex*^ = |*l*_*y*_−*L*_*y*_/2|. In all clusters the electron density is identical and given by *n*_*e*_ = 2.133 × 10^18^m^2^, and *γ* = 2.0 *e*V.

Figure 2 shows the transverse-mode (TM) and longitudinal-mode (LM) plasmon in atomic cluster system, where logatithmic coordinate is adopted for the *y* axis due to very large values of the energy absorption. According to the refs^12–15,24^, the energy-absorption peaks induced by the longitudinal electric field (see red lines) represent LM plasmon. On the other hand, the energy-absorption peaks induced by transverse electric field (see black lines) represent TM plasmon. In Fig. 2(a), the main LM plasmon (the one with the largest energy absorption) and the sub-main LM plasmon (the one with the second largest energy absorption) shift red with the increase of the longitudinal size, due to reduction of the energy gaps involved in the excitation in large size cluster system. However, the main and sub-main TM plasmon shift blue with the increase of the longitudinal size, which is similar to the behavior of one-dimensional end TM plasmon^13,14^. Since the external electric fields in Fig. 2(a) are linear, only dipole plasmons can be excited, so the plasmons shown in Fig. 2(a) are longitudinal dipole plasmon (LDP) and transverse dipole plasmon (TDP), respectively. Apart from the usual LDP and TDP, longitudinal quadrupole plasmon (LQP) and transverse quadrupole plasmon (TQP) are also illustrated in Fig. 2(b). It is clear that TQP is excited by the transverse symmetry electric field *V*_*T*_^*ex*^ = |*l*_*x*_ − *L*_*x*_/2|, while LQP by the longitudinal symmetry electric field *V*_*L*_^*ex*^ = |*l*_*y*_ − *L*_*y*_/2|. In Fig. 2(a,b), the frequency of the main quadrupole plasmon is larger than the one of the main dipole plasmon, it is agreed with the experimental result in refs^25,26^ that the quadrupole resonance are corresponding to higher energy excitation. However, the size dependence of LDP and LQP are similar, the size dependence of TDP and TQP are similar too. While the longitudinal size is very large, the frequencies of the main TDP and TQP have certain values, such behaviour is in agreement with the experimental observation of plasmon in graphene monolayer in the ref.^25^.

**Figure 3:**
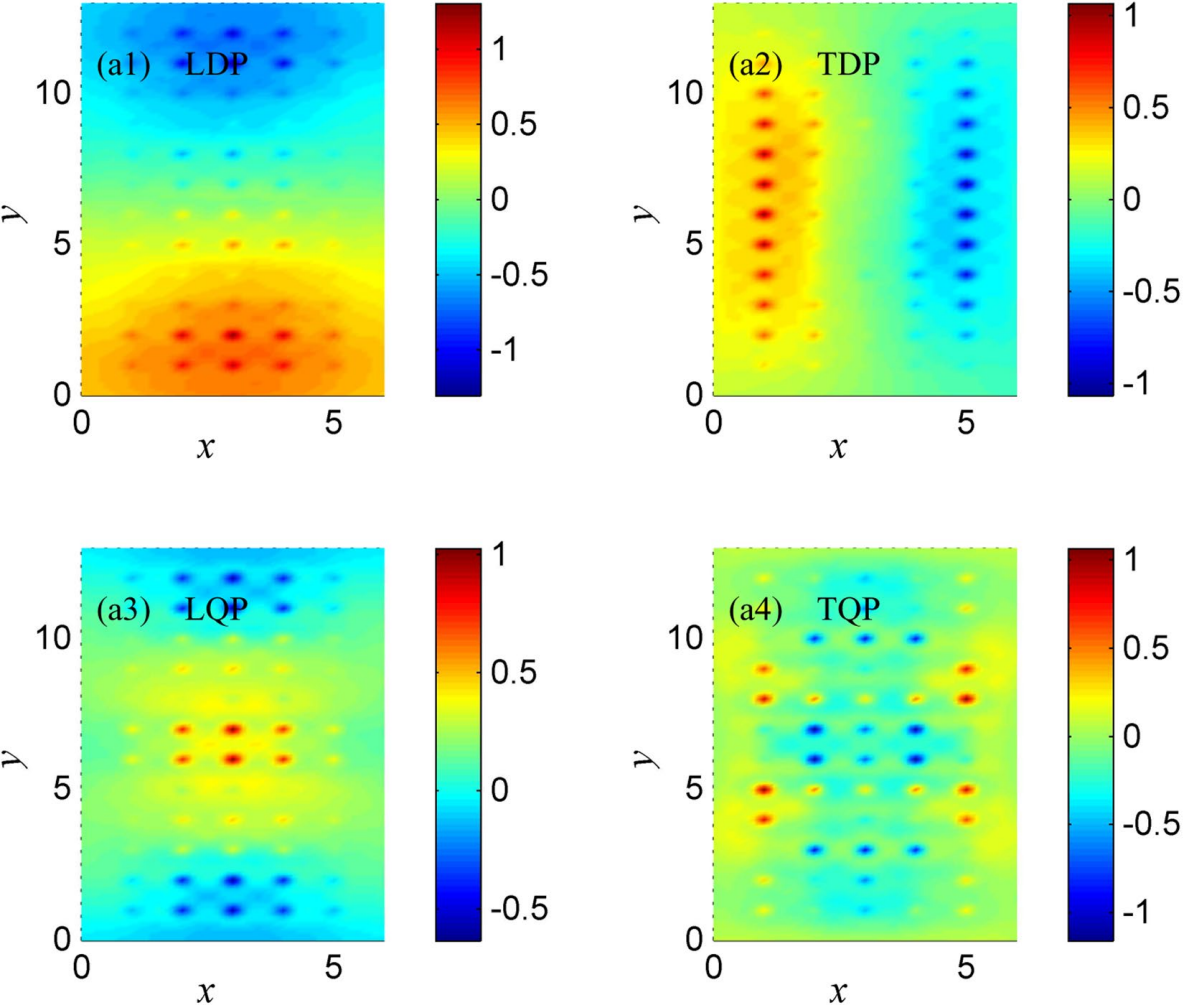
The charge distributions at the main (**a1**) LDP, (**a2**) TDP, (**a3**) LQP, and (**a4**) TQP. The specific LDP, TDP, LQP, TQP are marked with arrows for system 5 × 12 involved in Fig. 2, and the corresponding external fields are the same as that in Fig. 2.

The charge distribution, which is defined by $Q({\boldsymbol{r}},\omega )={\sum _{{\bf{l}}}Q({\boldsymbol{l}},\omega )|\varphi ({\boldsymbol{l}},{\boldsymbol{r}})|}^{2}$ with *ϕ*(***l***, ***r***) the *s*-wave function set to be $\varphi ({\boldsymbol{l}},{\boldsymbol{r}})={R}_{40}({\boldsymbol{r}}-{\boldsymbol{l}}){Y}_{00}(\theta ,\varphi )$, at the main LDP, TDP, LQP, and TQP is presented in Fig. 3. The 4 *s* orbit wave function is used here, so the calculation results are suitable for artificial one-layer systems consist of K Ca, Cu or Zn atoms, the valance electrons of which are in the 4 *s* orbit. In fact, for one-layer systems consist of Rb, Sr, Ag, Cd, Au, or Hg atoms, the results are similar, due the valance electrons of these atoms are all in the *s* orbit. At the main LDP and TDP (see Fig. 3(a1,a2), respectively), it shows dipole character along the longitudinal and transverse directions, respectively, whereas at the main LQP and TQP (see Fig. 3(a3,a4), respectively), it manifests symmetric character along the longitudinal and transverse directions, respectively. Moreover, the charge at the main TQP also show symmetric character along the longitudinal directions, implying that this TM plasmon can be both excited by transverse and longitudinal symmetry electric fields, as shown by the two energy-absorption peaks at the main TQP in Fig. 2(b). The characteristics of the TM plasmon excited by longitudinal electric field also happens in the systems *N*_*y*_ = 3, 6 and 12 in Fig. 2(a), due to the fact that TM plasmon may have dipole character along longitudinal direction.

**Figure 4:**
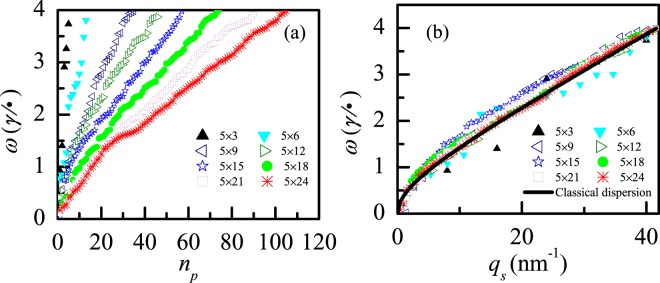
(**a**) The evolution of frequency spectra of LDPs with size. Here *n*_p_ is the number of the plasmon, and given by *n*_p_ = 1, 2, 3, ……, *N*_*p*_, representing that the plasmons are ordered from the lowest to the largest frequency, with *N*_*p*_ the number of the largest-frequency plasmon which is also the total amount of plasmons; (**b**) The evolution of plasmon dispersion with size for LDPs (scatter symbols), and the dispersion of classical 2D plasmon (continuous line). The corresponding clusters are the same as that in Fig. 2.

Normally (or Usually), only the main and sub-main plasmons are interested. However, it is important to take into account the low energy-absorption plasmons in the investigation of the frequency spectra of 2D plasmons, since the low energy-absorption plasmons and the high energy-absorption plasmons together form the spectra of plasmon. In the range of 0–4 ($\gamma /\hslash$), after all the peak frequencies of LDPs in Fig. 2(a) are collected, the frequency spectra of the LDPs is illustrated in Fig. 4(a). In clusters with less than 45 atoms (see the clusters with size smaller than 5 × 9), the total amount of LDPs is small, and the frequency spectra of LDPs is discrete. As the number of atoms is increased, more LDP modes come to appear in the larger-size systems due to more coupling ways of electrons. Such behavior of plasmon is somewhat analogous to the that of phonon (the quantization of lattice vibration). In addition, with the increase of the atoms, the gaps between plasmon frequencies become smaller, the frequency spectra turn to continuous, and the frequency spectra is decreased. All these changes are mainly caused by the fact that in larger-size system the energy levels near the Fermi energy are denser and thus closer to each other.

In order to gain a deeper understanding of the size effect of plasmon, we illustrate the plasmon dispersions of different-size clusters in Fig. 4(b). And our results are compared with the classical 2D plasmon dispersion ${\omega }^{2}=(\frac{2\pi {n}_{e}{e}^{2}}{{m}_{e}}){q}_{s}+\frac{3}{4}{{q}_{s}}^{2}{{v}_{F}}^{2}$^27^, where, *n*_*e*_ the face electron density, ${v}_{F}=\hslash {k}_{F}/{m}_{e}$ is the Fermi speed, and *k*_*F*_ the Fermi vector which satisfies *k*_*F*_^2^ = 2*πn*_*e*_. Quasi-wave vector *q*_*s*_ is introduced to study how the quantum mode transform to classical one. According to the plasmon frequency spectra, quasi-wave vector is defined by *q*_*s*_ = (*n*_*p*_/*N*_*p*_)*q_*max*_, where *q*_*max*_ is the maximum value of the wave vector which determined by the equation ${{\omega }_{\max }}^{2}=(\frac{2\pi {n}_{e}{e}^{2}}{{m}_{e}}){q}_{\max }+\frac{3}{4}{{q}_{\max }}^{2}{{v}_{F}}^{2}$, here, *ω*_*max*_ is the largest frequency of quantum-mode plasmon which equals to *ω*(*N*_*p*_) of the largest cluster.

For the cluster with few atoms, the quantum modes of plasmon are dispersed in the vicinity of the classical dispersion curve, as illustrated in Fig. 4(b). As the number of atoms is increased, the discrete quasi-wave vector gradually becomes continuous, and the discrete dispersion curve moves closer to the classic dispersion curve. For the cluster with more than 90 atoms (cluster size *N*_*x*_ × *N*_*y*_ ≥ 5 × 18), the dispersions of the quantum-mode plasmon coincide, to a large extent, with that of the classical plasmon. We argue that in this case the quantum mode of plasmon has transformed into the classical one. Such transformation is supported by the Bohr correspondence principle, which states that the behavior of quantum plasmon reproduces classical physics in the large size clusters.

**Figure 5:**
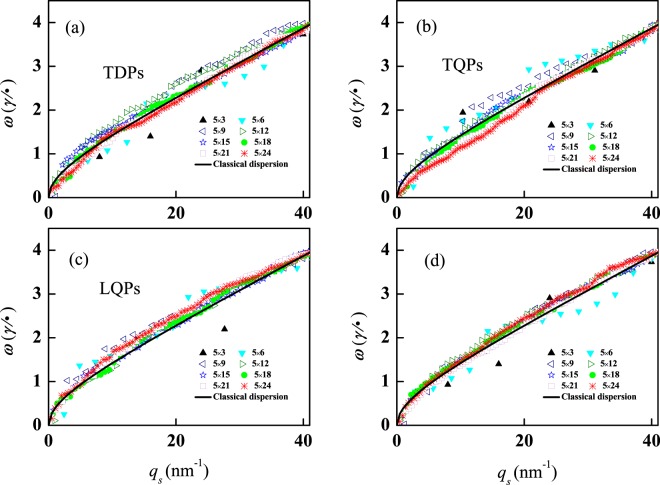
The evolution of plasmon dispersion with size. (**a**) for TDPs excited by *V*_*T*_^*ex*^ = *l*_*x*_; (**b**) for LQPs excited by *V*_*L*_^*ex*^ = |*l*_*y*_ − *L*_*y*_/2|; (**c**) for TQPs excited by *V*_*T*_^*ex*^ = |*l*_*x*_ − *L*_*x*_/2|; (**d**) for plasmons excited by *V*^*ex*^ = *l*_*x*_ + *l*_*y*_. The scatter symbols are obtained by the quantum-mode plasmon, and the continuous curves in (**a–d**) are obtained by the classical 2D plasmon which are the same to each others. The corresponding clusters are the same as that in Fig. 2.

The evolution of plasmon dispersion with size and subject to different external fields are presented in Fig. 5(a–d). In all these four subplots, the dispersion curves of the quantum-mode plasmons display a common feature: they become continous as the system size grows, and virtually transform into the same dispersion curve, i.e. the dispersion curve of classical 2D plasmon. Therefore the transform property of quantum-mode plasmon is independent on the external fields, as well-known, so does the classical 2D plasmon dispersion. It is worth noting that the main plasmon modes excited by different external fields (see Fig. 2) are not all the same, but they obey the same dispersion relationship in large clusters. So, if the dispersions of quantum-mode plasmon and 2D classical plasmon are coincident, the main plasmons can be found at the corresponding wave vectors in the classical dispersion curve.

**Figure 6:**
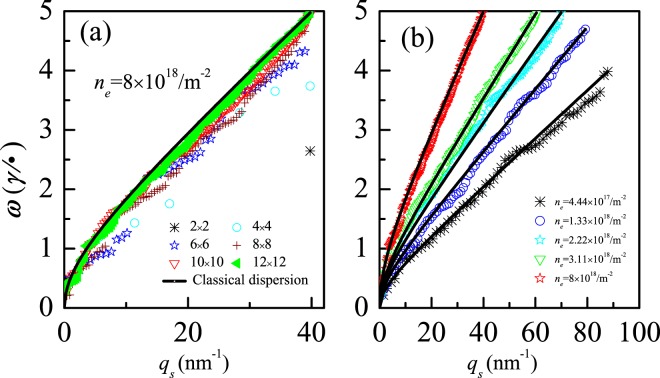
(**a**) The evolution of plasmon dispersion with size, where all plasmons are excited by *V*^*ex*^ = *l*_*x*_ + *l*_*y*_(scatter symbols), all clusters are square in shape and are the same in electron density *n*_*e*_ = 8 × 10^18^/m^2^; (**b**) The dispersion of plasmons that excited by *V*^*ex*^ = *l*_*x*_ + *l*_*y*_ (scatter symbols) for different electron density, where the cluster size is fixed to be 12 × 12, and *γ* = 3 *e*V. The electron density of the classical dispersion(continuous curves) equals the one of nearby scatter symbol.

In Figs 4 and 5, all clusters are rectangular in shape. Interestingly, for the square-shape clusters in Fig. 6(a), the transformation of plasmon from quantum to classical mode occurs, too, implying that the transformation property of plasmon is unaffected by the shape of 2D cluster. In Fig. 6(b), for the 12 × 12 cluster with different electron density, the dispersions of quantum-mode plasmon all agree well with that of the classical one. Moreover, the dispersion curve is higher for larger electron density, which indicates that the collective electronic excitations require larger frequencies in larger electron-density system due to more sparse energy levels around higher Fermi energy^12–15^.

## Conclusions

Based on the linear response theory, we have calculated the energy absorption of 2D atomic cluster systems. From the results various types of plasmon excited by electric potentials are specified, such as LDP, TDP, LQP and TQP. Furthermore, through the evolution of quantum-mode plasmon dispersion with size, the transformation property of quantum-mode plasmon is observed in the case of different external fields and in the clusters with different dimensions and different electron density, the transformation property shows a common feature that: more plasmon modes of plasmon exist in atomic cluster with more atoms, with the increase of cluster size, the discrete modes of plasmon will gradually evolve to the continuous one, and the dispersion curve of quantum-mode plasmon will transform into classical plasmon dispersion curve. Such property clarifies the size effect of plasmon in clusters with few atoms.
